# Comparison of national surveillance systems for Lyme disease in humans in Europe and North America: a policy review

**DOI:** 10.1186/s12889-022-13669-w

**Published:** 2022-07-07

**Authors:** Laurence Blanchard, Julie Jones-Diette, Theo Lorenc, Katy Sutcliffe, Amanda Sowden, James Thomas

**Affiliations:** 1 London School of Hygiene and Tropical Medicine, Faculty of Public Health Policy, London, WC1H 9SH UK; 2 University of York, Centre for Reviews and Dissemination, York, YO10 5DD UK; 3 Present address: School of Medicine, University of Nottingham, Nottingham, NG7 2RD UK; 4 University College London, UCL Social Research Institute, London, WC1H 0AA UK

**Keywords:** Lyme disease, Neuroborreliosis, Surveillance, Policy, Review, Europe, USA, Canada

## Abstract

**Background:**

Lyme disease incidence is increasing in Europe, the USA, and Canada. In 2010, a comparison of surveillance systems for Lyme disease (LD) in humans in 28 European countries showed that systems highly varied, making epidemiological comparisons difficult. Details by country were not published. In 2018, one of LD clinical manifestations, neuroborreliosis, was added under European Union (EU) surveillance to standardise definitions. In this study, we identified and compared, 10 years after the European inventory, the characteristics of national surveillance systems and policies for LD in humans, with additional countries.

**Methods:**

Thirty-four European and North American countries were included. Information on national “traditional” systems (which compile data reported by clinicians and laboratories) and “public participatory” websites and mobile applications (which collect information directly from the public) were searched in MEDLINE, a systematic evidence map, and Google. An existing framework on LD surveillance was adapted to capture information on the administration level, indicators, reporting entities, coverage, and obligation to report.

**Results:**

A surveillance system was found for 29 (85%) countries. Twenty-four had a traditional system alone, one had a public participatory system alone, and the remaining had both. Among countries with traditional systems, 23 (82%) administered them at the national level. Nineteen (68%) required mandatory reporting. Sixteen (57%) used both clinicians and laboratories as reporting entities. Eighteen (64%) employed case definitions, most of which considered both neuroborreliosis and erythema migrans (*n* = 14). Others monitored the number of positive laboratory tests and/or patient consultations. Public participatory systems were only implemented in countries employing either also sentinels or voluntary surveys, or no traditional system, suggesting their use as a complementary tool. Only 56% of EU countries had neuroborreliosis as an indicator.

**Conclusion:**

The situation remains similar to 2010 with persisting heterogeneity between systems, suggesting that countries prioritise different surveillance objectives for LD. Without a common indicator in Europe, it is difficult to get a clear epidemiological picture. We discuss four factors that potentially influence LD surveillance strategies: perceptions of severity, burden on resources, two-way communication, and the medical conflicts about LD. Addressing these with countries might help moving towards the adoption of common practices.

**Supplementary Information:**

The online version contains supplementary material available at 10.1186/s12889-022-13669-w.

## Background

Lyme disease (LD) is one of the most prevalent vector-borne diseases in Europe and the USA [1, 2]. It is transmitted to humans by the bite of ticks infected by a bacterium from the spirochete group *Borrelia burgdorferi* sensu lato. This study focuses on infections in humans. The most common clinical presentation is erythema migrans (EM), a cutaneous rash that can appear within the first days. Neurological manifestations such as Lyme neuroborreliosis (LNB) can develop later as well as musculoskeletal, cardiac, skin, and ocular conditions. LD has significant health-related quality of life implications and healthcare and societal costs [3].

Over the past 30 years, the incidence of LD has increased in Europe, the USA, and Canada, reaching particularly high rates in Central Europe and North-Eastern USA [1, 2]. Surveillance activities have become paramount to monitor trends and identify population groups at risk. At the European Union (EU) level, after several calls to standardise LD surveillance practices between countries, LNB and a case definition were added in 2018 to the EU list of communicable diseases for epidemiological surveillance [4]. Countries affected by LD were also pressed to make reporting obligatory [1]; prior sources had suggested that most did not [5, 6]. However, implementing the EU decision might require significant changes to attributes in some national surveillance systems and related laws.

Moreover, alongside the “traditional” surveillance systems noted above which compile data reported by clinicians and laboratories, “public participatory” (or “citizen-based”) surveillance systems are emerging [7]. In the latter, the general public directly reports the presence of symptoms or vectors, for instance on a website or mobile application (named hereafter “app”). While questions remain on data validity and sensitivity, these systems are seen as a low-cost option to complement traditional systems; for instance where their implementation is limited, or to capture individuals who do not use participating health services [7].

Knowing which indicator(s) national surveillance policies consider, and how they are reported, is crucial to understand and interpret data internationally. To our knowledge, the only inventory or comparison of surveillance systems for LD in humans was undertaken by the European Centre for Disease Prevention and Control (ECDC) in 2010 and focused on traditional systems. It consisted of an online survey which was then complemented by a literature search (no details provided). Thirty countries (27 EU and 3 in the European Free Trade Association (EFTA)) were invited through official channels to complete the questionnaire, of which 28 did. Data were aggregated at the EU/EFTA level and summarised in a conference report [8], but we were also given access to the details by country (personal communication with the ECDC in April 2022). The results showed that systems varied widely, especially among the indicators used.

The presence and clinical manifestations of the *Borrelia Burgdorferi* genospecies in Canada and the USA are different from those in Europe. Nevertheless, given the long-standing experience of the USA in monitoring LD, and the more recent yet well documented experience in Canada, potentially useful lessons can be drawn from these two countries for Europe from an organisational perspective. Thus, the objectives of this study were to 1. Map and compare, 10 years after the original inventory, the characteristics of national traditional and public-participatory surveillance systems and policies for monitoring LD in humans in EU and EFTA countries as well as in Canada and the USA; 2. Examine the use of LNB as an indicator within the EU. This project was part of a programme of evidence reviews on LD in humans that was commissioned by the Department of Health and Social Care, England, and registered on PROSPERO (CRD42017071515).

## Methods

Current national surveillance systems and policies for LD were included for 34 countries: Canada, the USA, the UK, the 27 EU countries as of 2021, and the four non-EU EFTA countries (Iceland, Liechtenstein, Norway, Switzerland). Research projects that are part of a national governmental surveillance strategy and involve periodical data collection were considered. The use of routine clinical data and clinical records was excluded. For pragmatic reasons, where systems vary between subnational areas, we used the national guidance when available and described the system overall at the national level.

Journal articles and government-related documents such as legislations, descriptions of systems, and surveillance reports were searched using three strategies: i) We scanned the studies on incidence and prevalence included in a systematic evidence map of research on LD produced as part of our overarching project [9]. ii) We searched MEDLINE (updated in July 2021) for studies published after the map (search strategy in Supplement A). iii) We searched the websites of relevant governmental authorities (e.g. national public health department) and via Google (search strategy in Supplement B). Google Translate was used when necessary. Information was rated as unclear when incomplete or where contradictions were found. Searches i and iii were conducted by one reviewer and verified by another in 2017 (JJD, LB and TL) and reconducted in September–October 2020 (LB).

Data were extracted by country into a framework in Excel by one reviewer (LB) and checked by a second (JJD). The framework for traditional systems (Table 1) was based on the five surveillance characteristics for LD suggested by van den Wijngaard et al. [6] with some modifications: administration level, key indicators, reporting entities, coverage, and obligation (Table 1). “Administrative level” was used to describe the level of power where systems are regulated and implemented like the ECDC did [8], rather than the level of precision of the data published. “Key indicators” focused on manifestations in humans only (not on the presence of humans in areas at risk and infection in wildlife and ticks). We divided these into three categories: case definitions, positive laboratory tests, and medical patient consultations. “Type of reporting” was renamed “Obligation” and focused on the obligation to report at the national level. Public participatory websites and apps were mapped separately by the type of information reported. Data were synthesized narratively by surveillance system characteristics based on the framework using descriptive statistics and examples. Results about Canada and the USA were compiled separately due to differences in genospecies. Ethical approval was not required since this is an analysis of published information.

**Table 1:** Characteristics of surveillance systems for Lyme disease in humans (framework for data extraction and analysis)

Characteristics	Definitions
**Traditional systems**	
Administrative level	The responsibility to regulate and implement the system lies with the authority at the: • National level; OR • Subnational level
Key indicators	Definition of what is recorded as “Lyme disease”. More than one could be used: • Use of a case definition for EM, LNB, and/or other “late” clinical manifestations (e.g. Lyme carditis or arthritis), and whether these are based on clinical signs only and/or confirmed with a laboratory test. Where several levels of confidence are used (e.g. probable, confirmed), the definition for confirmed cases was extracted • Positive laboratory tests; • Medical patient consultations for tick bites, EM or other manifestations
Reporting entity	Unit responsible for reporting a positive case to the system: • The clinician treating a patient with the disease; OR • The laboratory; OR • Both the clinician and laboratory, either in the same or different areas of the country
Coverage	The surveillance system: • Is comprehensive (all reporting units are invited or required to report data); OR • Uses samples of reporting units (e.g. sentinels) or other non-comprehensive methods
Obligation	The reporting of information at the national level is: • Mandatory (e.g. by law); OR • Voluntary; OR • It varies between areas
**Public participatory websites and apps**	
Indicators	The system collects information directly from the general public using a website and/or app. Indicators: tick bites, EM and/or other manifestations

Apps: mobile applications; EM: erythema migrans; LNB: Lyme neuroborreliosis

Framework adapted from [6]

## Results

Twenty-nine of the 34 countries have a national governmental surveillance system or policy for LD: 27 (84%) of the European countries assessed as well as Canada and the USA. All but Liechtenstein use at least one traditional system, i.e. data reported by clinicians and/or laboratories. Five have a public participatory website or app. The characteristics are summarized in Tables 2 and 3. Analyses of the traditional systems (*n* = 28) and public participatory systems (*n* = 5) are presented below.

**Table 2:** Characteristics of national surveillance systems for Lyme disease in humans by country (*N* = 34)

**Countries**	**Administrative level**		**Reporting entities overall**			**Coverage & Obligation**				**Participatory website/app** (general public)	**No national government-led system found**
	National	Sub-national	Both clinicians & labs	Clinicians only	Labs only	Comprehensive & mandatory at national level	Comprehensive & voluntary at national level	Voluntary samples	Other		
**EU COUNTRIES (*n* = 27)**											
Austria [10]											Except that it “requires monitoring” … depending on epidemiological situation” (translated from German)[10]. No further information or statistics found
Belgium [11]	X		X					Lab sentinels, clinician sentinels & national lab reference centre		X	
Bulgaria [12]	X		X			X					
Croatia [13]	X		X			X					
Cyprus											X
Czech Rep. [14]	X			X		X					
Denmark [15]	X		X			X^a^					
Estonia [16]	X			X		X					
Finland [17]	X				X	X					
France [18, 19]	X		X					Clinician sentinels & national lab reference centre		X	
Germany [20, 21]		X	Varies between states						Mandatory in 9/16 states		
Greece											X
Hungary [22]	X		X			X					
Ireland [23]	X		X			X					
Italy [24]	X			X		X					
Latvia [25]	X			X		X					
Lithuania [26, 27]	X			X		X					
Luxembourg [28]	X			X		X					
Malta											X
Netherlands [5]	X			X			Survey to all			X	
Poland [29]	X		X			X					
Portugal [30]	X		X			X					
Romania [31]	X			X		X					
Slovakia [32]	X		X			X					
Slovenia [33]	X		X			X					
Spain [34]	X				X			Lab sentinels			
Sweden											X
**Subtotal EU**	**21**	**1**	**12**	**8**	**2**	**17**	**1**	**3**	**1**	**3**	**5**
**OTHER EUROPEAN COUNTRIES (*n* = 5)**											
Iceland [35]	X		X			X					
Liechtenstein										(only system found)	
Norway [36]	X		X			X					
Switzerland [37]	X			X				Clinician sentinels		X	
UK [39–42]		X			X		X^b^				
**Subtotal Europe**	**24**	**2**	**14**	**9**	**3**	**19**	**2**	**4**	**1**	**5**	**5**
Canada [43, 44]		X	X				X				
USA [2]		X	X				X				
**TOTAL**	**24**	**4**	**16**	**9**	**3**	**19**	**4**	**4**	**1**	**5**	**5**

App: mobile application; EFTA: European Free Trade Association; EU: European Union; Lab: laboratory

^a^Denmark: The reporting is only mandatory for clinicians; however, laboratory data are reported automatically via the electronic system MiBa and contributes to surveillance activities

^b^UK: It is unclear whether all the laboratories that test for Lyme disease report data to the system in Northern Ireland

All categories are mutually exclusive

**Table 3:** Key indicators used at the national level in countries that monitor Lyme disease (*N* = 29)

Countries	Case definitions (*n* = 18)					Positive laboratory tests (*n* = 4)	Medical patient consultations (*n* = 3)		Public participatory website/app (*n* = 5)		Unclear / Not reported (*n* = 3) & Notes
	EM (clinical signs only^a^)	EM (clinical + lab^a^)	EM (others^a^)	LNB (clinical + lab)	Other (clinical + lab)		Tick bites + EM	Other “late” clinical manifestations	Tick bites	Clinical manifestations	
**EU COUNTRIES THAT HAVE A POLICY OR SYSTEM (*n* = 22)**											
Belgium [11]						X	X		X	X	
Bulgaria [45]		X		X	X						
Croatia [46]		X		X	X						
Czech Republic [14]		X		X	X						
Denmark [15]				X							
Estonia											Unclear / No national definition found (legislation only specifies ICD-10 code A69.2)[16]
Finland [17]						X					
France [18]	X			X	X				X		
Germany [20]	X			X	X						In at least 9/16 states
Hungary [22]	X			X							
Ireland [23]				X							
Italy											Unclear / No national definition found
Latvia [25]				X							Refers to the list of EU case definitions, so LNB
Lithuania											Unclear / No national definition found (legislation only specifies ICD-10 code A69.2)[26]
Luxembourg [47]			Unclear if lab tests required	X							
Netherlands [5]							X		X	X	
Poland [48]	X			X	X						
Portugal [30]				X							
Romania [31]		X		X	X						
Slovakia [32, 49]		X		X	X						Cases clearly reported using ICD-10 codes A69.2, G63.0 and M01.2 [49]
Slovenia [50]		X		X	X						
Spain [34]						X					Some autonomous communities also have a case definition
**Subtotal EU**	**4**	**6**	**1**	**15**	**9**	**3**	**2**	**0**	**3**	**2**	
**OTHER EUROPEAN COUNTRIES (*n* = 5)**											
Iceland [51]											No clinical nor lab information required
Liechtenstein									X		
Norway [36]		Multiple EMs only		X	X						
Switzerland [37]							X	X	X		
UK [38]						X					
**Subtotal Europe**	**4**	**7**	**1**	**16**	**10**	**4**	**3**	**1**	**5**	**2**	
Canada [52]		X		X	X						Some provinces record EM without lab
USA [53]			Confirmed with exposure in high incidence state or with lab and exposure in low incidence state	X	X						
**TOTAL**	**4**	**8**	**2**	**18**	**12**	**4**	**3**	**1**	**5**	**2**	

App: mobile application; EFTA: European Free Trade Association; EU: European Union; EM: erythema migrans; Lab: laboratory; LNB: Lyme neuroborreliosis

^a^ The three EM categories are mutually exclusive

No government-led system or policy was found in Austria, Cyprus, Greece, Malta and Sweden

### Traditional surveillance systems

#### Administration level

Among the 26 European countries with a traditional system, 24 (92%) administer it at the national level [2, 5–11, 18–22, 26–28, 33, 34, 34]. This includes Spain where LD surveillance is a regional responsibility [36] because we focused on their national sentinel system [37]. In the remaining two European countries (Germany, the UK) as well as Canada and the USA, responsibility lies at subnational level (i.e. state, country, province, or territory). Nevertheless, all have some degree of national governance. For instance, LD has a nationally notifiable status in Canada and the USA. A national case definition was found for Canada [43], Germany [51] and the USA [52]. All subnational areas in Canada, the UK, and the USA submit data to a national authority, which publishes them [53, 54, 55].

#### Key indicators

The categories of indicators in traditional surveillance systems included the number of 1) cases, 2) confirmed laboratory tests, and 3) patient consultations. Sixteen (62%) of the 26 European countries with a traditional system as well as Canada and the USA employ case definitions (explicit criteria for categorising a positive case of LD, typically specifying clinical manifestations with or without laboratory confirmation) [14, 15, 18, 20, 22, 23, 25, 30–32, 36, 45–48, 50, 52, 53]. Croatia, Czech Republic, Romania and the USA are examples of countries that have developed extensive definitions: cases are classified as suspected, probable, or confirmed, with clearly defined clinical presentation, laboratory confirmation and/or exposure [14, 31, 46, 23]**.** On the other hand, we did not find case definitions for Slovakia but cases in national surveillance reports are clearly reported using codes A69.2, G63.0 and M01.2 from the International Classification of Diseases (ICD-10) by the World Health Organization [30], which refer to different manifestations of LD. All 16 European countries employing case definitions consider LNB: 10 together with EM and other late manifestations, two with EM, and four LNB alone. Fifteen are part of the EU, meaning that 56% of EU member states monitor LNB using a case definition. Since the EU decision in 2018, some countries have integrated the EU case definition into their own, e.g. Bulgaria, Croatia, Ireland, Poland, Portugal and Romania [31, 45, 46, 48, 49, 53]. All but Croatia already had LNB in their case definitions. Canada and the USA consider both EM and late manifestations including LNB.

Other indicators employed in Europe include positive laboratory tests alone (Finland, Spain, UK) [17, 34]; number of patient medical consultations for tick bites and EM (the Netherlands, Switzerland) [5, 37]; and both positive laboratory tests and patient consultations (Belgium) [11]. Neither clinical nor laboratory information is required to report cases in Iceland [51]. We were unable to find clear national indicators for Italy, Estonia, and Lithuania. Only the Netherlands [3] and Switzerland [37] appear to monitor chronic aspects of LD.

#### Reporting entities

Fourteen (54%) of the 26 European countries with a traditional system as well as Canada and the USA use both clinicians and laboratories as reporting entities [2, 11–5, 11, 13–14, 15, 16, 17, 18, 20, 22, 23, 24, 28, 29]. Data can be reported separately or compiled by the authority in charge. Belgium is an example where data are published separately for four different systems: i) periodical surveys from general practitioner (GP) sentinels; ii) monthly reports about laboratory sentinels; iii) yearly reports from the “National Reference Centre for Borrelia burgdorferi” (translated from French); and iv) a participatory website and app [30]. In Norway, by contrast, information from clinicians and laboratories are aggregated into a single database by the Norwegian Institute of Public Health using personal identification numbers. When both clinical signs and a positive laboratory test match, they are registered as a single case [31]. Of the remaining European countries, nine (35%) only use clinicians [32, 33, 34, 35–36, 36, 37] and three use only laboratories (Finland, Spain, UK) [43, 44].

#### Coverage and obligation

Twenty-one (81%) European countries with a traditional system as well as Canada and the USA use a comprehensive coverage strategy, meaning that every individual or entity corresponding to the systems’ reporting body profile are included in the system, e.g. all laboratories and/or all clinicians. Most have made reporting mandatory at the national level (*n* = 19) [12–17, 22–26, 28–33, 35, 36]. Canada, the UK, and the USA require voluntary reporting at the national level; however in practice, all subnational areas transmit data. The Netherlands uses a unique comprehensive and voluntary system consisting of retrospective surveys sent to every GP at 4–5-year intervals [5].

By contrast, Belgium, France, Spain, and Switzerland use samples (sentinels) at the national level. Sentinels are samples of reporting units that are voluntary registered in a network and trained to actively report information to the system. They are generally selected to represent the general population or for their higher likelihood to see cases [6]. France, Switzerland, and Belgium use clinician sentinels that either regularly report cases (France, Switzerland) [18, 37] or complete prospective surveys every 4–6 years (Belgium) [11]. Furthermore, both Belgium and Spain use laboratory sentinels [11, 34]. There is no country-wide reporting system for LD in Germany but some states employ a mandatory approach [20].

#### Public participatory surveillance systems

In Belgium, France, Liechtenstein, the Netherlands, and Switzerland, a national governmental authority is involved in a public participatory website or app (details in Supplement C). The general population is invited to report tick bites (all countries) as well as signs of EM after a tick bite (Belgium, the Netherlands), and fever or “‘other forms” of LD (the Netherlands). Liechtenstein and Switzerland share a joint system.

## Discussion

To our knowledge, this review provides the first publicly available international comparison of human LD surveillance systems with details by country. It updates data collected by the ECDC in 2010 which were aggregated at the EU/EFTA level in a conference report [8]. Twenty-seven (84%) of the 32 European countries included as well as Canada and the USA have a national surveillance system or policy for LD in humans. Of the 32 European countries, more than half require mandatory reporting at the national level, which is more than the “few” recently suggested [17]. However, two years after the EU announcement, only 56% of EU countries consider LNB. This is without considering the specific EU clinical and laboratory criteria, so the proportion using the EU case definition is likely to be smaller. Based on our personal observations (not a systematic assessment), countries that employ the EU case definition already had a mandatory and comprehensive system with a case definition. Considering the spread of the disease, health and healthcare impacts, growing awareness and pressure from health professionals and the public, and following the EU leadership, we would have expected more countries to monitor the disease and standardise practices.

Regarding EU/EFTA countries, when comparing the findings from the 2010 survey by the ECDC (*n* = 28 including the UK) [8] to those from our study (*n* = 31, excluding the UK since it had since left the EU), our results suggest that the situation has not changed much. Using the data shared by the ECDC (personal communication in April 2022), we can say that the additional countries in 2020 were Croatia, Italy, Liechtenstein and Luxemburg. A similar proportion of countries have a traditional system (*n* = 23 [82%] in 2010 vs 25 [81%] in 2020) although they slightly differ: we found a system for Ireland (it had none in 2010) and for three of the four additional countries (Croatia, Italy and Luxemburg) but not for Austria while it was listed in 2010 as having a sentinel, and we excluded the UK system. A few more countries use a comprehensive system (17 [61%] in 2010 vs 20 [65%] in 2020) including Ireland, Croatia, Italy and Luxemburg. Contrary to the ECDC in 2010, we also added the Netherlands to this list but not Germany, suggesting differences in interpretation for these systems (which are different from the norm), and we excluded the UK again. The number of countries using mandatory reporting increased from 16 [57%] in 2010 to 20 [65%] in 2020), once again due to the addition of Ireland, Croatia, Italy and Luxemburg. In brief, the increase in the proportion and number of countries employing a comprehensive and mandatory system was mainly due to Ireland now having one, Croatia joining the EU, the UK leaving with a voluntary system, and the inclusion of additional countries, rather than multiple previous countries having changed their approach. Results for the reporting units and indicators cannot be compared due to differences in focus and categories.

The persistent high heterogeneity across the systems, particularly for the types of indicators (including the case definitions themselves) and reporting entities, suggests that governments seek different surveillance objectives. While there is no perfect method (e.g. all are prone to under- or over-reporting [6, 8, 44, 56], and different reporting entities and obligation levels give different results [57]), using a common indicator in Europe would at least ensure that data are collected about the same thing. The diversity of indicators used is nevertheless not surprising given the absence of medical consensus on the definition of LD symptoms and their diagnosis [1, 3]. Differences in healthcare systems and data logistics have also been highlighted [6]. Drawing from the European and North American literature retrieved in our searches, in the following we suggest four additional factors which are not directly linked with specific *Borrelia Burgdorferi* genospecies and potentially influence national LD surveillance strategy decisions: perception of severity, burden on resources, two-way communication, and the medical conflicts about LD.

### Perception of severity

Since LD is not communicable between humans and rarely leads to outbreaks, it has been argued that there is a lesser need to identify every single case [8, 20]. This was one of the reasons for the ECDC to suggest LNB as the sole indicator for the EU [8]. In France, GP sentinels were chosen since LD causes few deaths and hospitalisations [58]. However, the limited sensitivity of LNB alone and sentinels [6] might explain why few countries have chosen these options. By contrast, in Canada, LD is emerging and rapidly expanding to new regions. To understand the situation, calls have been made to expand the scope of surveillance.

### Burden on resources

Balancing data precision with resources availability is a challenge from which LD surveillance is not spared. On the one extreme, Iceland uses automatic submissions from medical records without clinical nor laboratory information. Data have been said to be so unreliable that it is unclear whether the disease is present in the country or not [51]. At the other extreme, in American states where incidence rates are extremely high, surveillance staff question whether “intensive statewide” LD surveillance is practicable [15, 53]. Different strategies have been implemented to reduce the workload. In Connecticut, cases with missing information are directly considered as lost to follow-up [56]. In Maryland, some local health departments only investigate cases that meet the laboratory criteria [56]. At the national level, laboratory tests are only required to confirm EM cases in low-incidence states (< 10 confirmed cases per 100,000 inhabitants) [57]. The combination of voluntary reporting with public participatory systems found in our study suggests this as another strategy to improve data precision while limiting the burden on resources. Research is needed to assess whether this is effective. Using automated laboratory data like in Denmark is another avenue for reducing the resources needed [57].

### Two-way communication

Public participatory surveillance systems can be seen as an opportunity to kill two birds with one stone; to gather data and educate the public at the same time. This is the case for the Dutch app, which was designed as a “two-way communication” tool [7]: the public can both provide and receive information. However, an evaluation showed no difference in knowledge and intention to apply preventive strategies between the intervention and control group [59]. More research is needed, including between different population groups.

### Medical conflicts about LD

The limited adoption of the EU case definition and monitoring of chronic LD perhaps reflects the two-decades long debates about LD, which started in the USA and has expanded to other countries including France and the UK [58, 60]. On the one hand, according to the Infectious Diseases Society of America (IDSA), whose guidelines are endorsed by the US-CDC, there is no evidence for chronic LD, and post-treatment LD is uncommon. On the other hand, patient groups and ‘Lyme-literate physicians’ including the International Lyme and Associated Diseases (ILADS) frequently diagnose and treat these. These disputes are said to keep clinicians and researchers on the defensive and to limit their engagement with the disease, which hinders knowledge and patient care [3]. A systematic review on the experience of LD diagnosis produced as part of our project showed that patients with persisting symptoms perceived clinicians as ambivalent, sceptical, or condescending [3]. Our follow-up consultation on these findings with patients and clinicians found that some clinicians feared to be vilified by colleagues, tracked by authorities, or to lose their license if they went beyond the guidelines [3]. Could this climate also affect the choice of surveillance strategies and explain the relative status quo in the policies? Assessing the views and experiences of decision-makers involved in LD surveillance could help understand whether these conflicts partly explain the moderate engagement with the EU case definition and absence of national surveillance policy in some countries that have high incidence rates.

Strengths of our review include comprehensive searches, data extraction performed by one researcher and verified by a second, and the use of a published theoretical framework which we adapted to guide the system classification and synthesis of findings. Although a range of surveillance characteristics were documented, others could have been considered. For instance, we did not differentiate the types of laboratory tests and clinical signs used for each manifestation. Governmental agencies were not contacted to validate data or obtain further information. We did not assess systems in terms of efficiency, sensitivity, acceptability, data quality, level of aggregation of data, nor through time.

## Conclusions

This review highlights that high heterogeneity persists between European national surveillance systems and policies for human LD despite the introduction of an EU case definition. Without a common indicator, it is difficult to get a clear epidemiological picture. As LD continues to spread, understanding the factors that influence national LD surveillance strategies and discussing them with countries might support the adoption of common practices within the EU and the European continent.

## Supplementary Information

**Additional file 1. **

## Data Availability

All data generated or analysed during this study are included in this published article.

## Abbreviations

App: Mobile application
EM: Erythema migrans
EFTA: European Free Trade Association
ECDC: European Centre for Disease Prevention and Control
EU: European Union
Lab: Laboratory
GP: General practitioner
LNB: Lyme neuroborreliosis
