# Supplementary material for: Comparison of national surveillance systems for Lyme disease in humans in Europe and North America: a policy review
Source: BMC Public Health. 2022 Jul 7;22:1307. doi: 10.1186/s12889-022-13669-w (PMC9264653; doi:10.1186/s12889-022-13669-w)
Supplement: Supplementary file 1 — Additional file 1. [file 12889_2022_13669_MOESM1_ESM.docx]

# Additional material

## **A. Literature search strategy for the journal articles published after the systematic evidence map (i.e., from 2016)**

Database: MEDLINE(R) ALL, from 1946 to July 21 2021 (Ovid).

Search date: 22-07-2021 (with the use of a filter limiting the search from 2016 to 21 July 2021)

| **Search terms** |
| --- |
| surveillance.mp. or exp Public Health Surveillance/ or exp Behavioral Risk Factor Surveillance System/ or exp Population Surveillance/ or exp Sentinel Surveillance/ |
| exp Epidemiological Monitoring |
| exp Public Policy/ or policy.mp. or exp Health Policy/ or exp Policy/ or exp Policy Making/ |
| exp Prevalence/ or prevalence.mp. |
| exp Incidence/ or incidence.mp. |
| lyme disease.mp. or exp Lyme Disease/ |
| borreliosis.mp. or exp Borrelia Infections/ |
| borrelia burgdorferi.mp. or exp Borrelia burgdorferi/ |
| borrelia.mp or exp Borrelia/ or exp Borrelia burgdorferi group/ |
| erythema migrans.mp. or exp Erythema Chronicum Migrans/ |
| exp Lyme Neuroborreliosis/ or neuroborreliosis.mp. |
| ((borreli* or lyme) adj2 (arthritis or carditis)).mp, |
| 1 or 2 or 3 or 4 or 5 |
| 6 or 7 or 8 or 9 or 10 or 11 or 12 |
| 13 and 14 |
| limit 15 to yr="2016 - Current" |

## **B. Search strategy used in Google**

For each country, the following strategy was used in Google in that order until relevant, reliable, and up-to date information was found. The searches were conducted firstly in English, then in the country’s official language when different from English and when no relevant, reliable, and up-to date information could be found:

1. Country name AND surveillance AND (“Lyme disease” OR borrelia OR neuroborreliosis OR burgdorferi)
2. Country name AND “notifiable diseases” AND (Lyme OR borrelia OR neuroborreliosis OR burgdorferi)
3. Country name AND (notifiable OR notification) AND diseases
4. Country name AND (statistics or report) AND “infectious diseases”

## **C. Additional information on public participatory surveillance systems**

| **Country** | **System’s name** | **Launch year** | **References** |
| --- | --- | --- | --- |
| Belgium | Tick Net | 2015 | Sciensano [Belgium]. Sciensano & Lyme disease 2018. https://www.sciensano.be/en/health-topics/lyme-disease/role (accessed 26 September 2021) |
| France | CiTIQUE | 2017 | INRAE Nancy [France]. Citique 2020. https://www.citique.fr/ (accessed 26 September 2021) |
| Liechtenstein (system shared with Switzerland) | Tick | 2016 | ZHAW University. Tick prevention with your smartphone (undated). https://www.zhaw.ch/en/lsfm/business-services/institute-of-natural-resource-sciences/ticks/tick-app/ (accessed 26 September 2021) |
| The Netherlands | Tekenradar | 2012 | Wageningen University,, Wageningen University, RIVM en De Natuurkalender. Tekenradar.nl 2020. https://www.tekenradar.nl/ (accessed 26 September 2021) |
| Switzerland | See Liechtenstein | | |
